# Behavior, Welfare, and Meat Quality of Japanese Black Cattle Under Different Transportation Methods

**DOI:** 10.1111/asj.70082

**Published:** 2025-07-16

**Authors:** Gianne Bianca P. Manalo, Mitsushi Kobayashi, Jitsuo Mizowaki, Masayuki Nagase, Makoto Iwamoto, Kenta Koike, Shigeru Ninomiya

**Affiliations:** ^1^ The United Graduate School of Agricultural Science Gifu University Gifu‐shi Gifu Japan; ^2^ JA Hida Meat Takayama Gifu Japan; ^3^ Faculty of Applied Biological Sciences Gifu University Gifu‐shi Gifu Japan

**Keywords:** behavior, Japanese Black cattle, transportation, welfare

## Abstract

Despite extensive research on livestock transportation, the specific effects of different transportation methods on the behavior, welfare, and meat quality parameters of Japanese Black cattle (JBC) remain unclear. This study is the first comprehensive investigation comparing two transport methods: same farm (SF) and multiple farm (MF), while accounting for combined factors such as distance and introduction of unfamiliar animal along the journey. A total of 42 JBC steers (784 ± 81.3 kg) from three production farms (Farms A, B, and C) were observed using focal sampling at a slaughter facility. Generalized linear models (GLMs) were used to analyze the effects of two transportation methods. Results showed that cattle transported via the MF method exhibited significantly higher frequencies of drinking (*p* = 0.005) and eating (*p* = 0.005), suggesting increased fatigue. Additionally, self‐grooming behavior was significantly higher in Farm B under the SF method (*p* = 0.002). No significant differences in meat quality parameters were observed between the two transportation methods. These findings suggest that transporting animals from SF method may help reduce transport‐related stress upon arrival at the slaughter facility. Further investigation is needed to explore the physiological and economic implications of different transportation methods.

## 1 Introduction

Livestock transportation is an essential component of the meat industry, but it poses significant challenges for animal welfare. In fact, this is one of the most critical phases of the animals which may directly affect their efficiency, productivity as well as their well‐being (Gebresenbet et al. 2012; Hemsworth et al. 2011; Ljungberg et al. 2007). Despite under optimal conditions, transportation can result in injury, discomfort, exhaustion, anxiety, and suffering to animals (Özdemir et al. 2022). Additionally, an increased incidence of lameness, nonambulatory animals, and mortality was observed during this period (González et al. 2012a).

On average, cattle are transported four to six times throughout their lifespan, moving from cow‐calf operations to feedlots for finishing, and finally to slaughterhouses (González et al. 2012b; Schuetze et al. 2017). The centralization of livestock facilities necessitates more frequent and longer transport durations, particularly to slaughterhouses. To improve transport conditions for cattle, several studies have investigated in relation to its different factors, including transport duration (Meléndez et al. 2020; Ferreira et al. 2006), microclimate (Stanford et al. 2011; Goldhawk et al. 2014), space allowances (Eldridge and Winfield 1988; González et al. 2012c), stocking density (Abubakar et al. 2021; Tarrant et al. 1988), trailer design (Lambooij et al. 2012), and stockmanship (Yost et al. 2020; Probst et al. 2013).

Although livestock transportation has been widely explored, this study is the first to investigate the impact of common cattle transport methods at a slaughter facility. Specifically, the same farm (SF) method, which involves direct transport from a single production farm, and multiple farm (MF) method, which includes an additional stop at another production farm to pick up one more steer before reaching the slaughter facility. Previous research has assessed the effects of transport duration and distance on body weight and blood profiles in Japanese black heifers (Hayashi 2017), however, direct impact on behavior, welfare and meat quality of Japanese black steers remain unclear. Moreover, existing research has only focused in transit duration as a primary factor. This study will assess confounding factors such as transport distance and the introduction of unfamiliar animals during transit.

This study hypothesizes that cattle transported from MFs will exhibit higher levels of fatigue and stress, which will manifest in their behavior. By analyzing the consequences of this method, the research seeks to provide a comprehensive understanding of how this transportation approach affects cattle welfare, offering insights that can guide improvements in handling practices to ensure better outcomes for the animals. Additionally, this research addresses the increasing public concern regarding ethical food production practices, highlighting the significance of humane handling methods.

## 2 Materials and Methods

### 2.1 Animals

Forty‐two (42) Japanese black steers were selected from the list of transported animals at the JA Hida Meat slaughter facility located in Yokamachi, Takayama City, Gifu Prefecture. The selection was conducted between March 31, 2022, and May 25, 2023. The steers had a mean age of 28.7 months, an average body weight of 784 ± 81.3 kg, and an average fattening period of 19 ± 1.9 months.

### 2.2 Experimental Design

Because the authors conducted the study at a slaughter facility, they did not have access to detailed information regarding the rearing conditions at the farm level. To address this limitation, a stratified sampling strategy was employed.

Specifically, three production farms (Farms A, B, and C) were purposefully selected. From each farm, an equal number of animals (*n* = 14) were sampled for each transport method, resulting in a balanced representation across both transport groups.

### 2.3 Transportation Methods

Two transportation methods were used in this study to compare their effects on Japanese Black cattle (JBC).

- SF method**—**animals were collected by a transport vehicle from their production farm and transported directly to the slaughter facility.
- MF method**—**animals were collected by a transport vehicle from their production farm, then the vehicle stopped at other farm to pick up one additional steer before proceeding to the slaughter facility.

### 2.4 Transport Conditions

In this study, each transport event involved five to six steers per vehicle. They were delivered to the slaughter facility between 11:00 a.m. and 2:00 p.m. The additional steer in the MF group were picked up along the same route going to the slaughter facility, after the truck left the first production farm**.** Because of this, the transport distance differences between SF and MF groups from each production farm were minimal, ranging from 0.1 to 3 km. The transport duration was expected to be longer in MF groups, since they collected additional steer in another production farm. The estimated distance of each transport method across different production farms are presented in Table 1.

**TABLE 1:** Estimated distance (km) of transport methods across different production farms.

Production farm	Distance (km)
Farm A	
Same farm	17.1
Multiple farm	19.9
Farm B	
Same farm	44.1
Multiple farm	44.2
Farm C	
Same farm	112
Multiple farm	115

The distance from the production farms to the slaughter facility were estimated using address information of each farm from the search service of Gifu prefecture or Google and verified by Google Maps.

### 2.5 Environment of the Slaughterhouse

At JA Hida Meat's holding facility, there were 70 stalls, each 80 cm wide, made with iron partitions (height 105 cm, depth 120 cm). Each stall was equipped with a feed and a water trough. There was no bedding, and the floor was concrete. Each selected animal was individually confined to a stall from arrival until the following day of slaughter. During confinement, feed (Fusuma A feed, produced by Nisshin Flour Milling Co. Ltd.) was provided twice a day, in the afternoon of the arrival day and the morning of the next day, with approximately 200 g per feeding. Additionally, water was continuously available from the tanks.

### 2.6 Behavioral Parameters

Preliminary observations were done before the actual study to finalize the behaviors listed in the ethogram. Antagonistic behavior was removed since this behavior is not consistently observed. Below (Table 2) are the list of behaviors and postures that used in observing JBC in this study.

**TABLE 2:** Ethogram used in this study in observing JBC behavior.

Behaviors	Definition
Drinking	Puts mouth or tongue on the water surface of the tank
Eating	Puts mouth inside the feeder and licking it with their tongue
Ruminating	Regurgitates previously consumed feed and chews it further in a standing and lying position
Resting	The animal remains stationary in a standing or lying position, showing no active movement.
Others	Behaviors not mentioned above
Sniffing the ground	Stretches the neck towards the ground, swinging the head
Allogrooming	Stretches the neck towards other cattle and licking it
Self‐grooming	Bites or scratches itself using hoofs or leaning against the structures of the slaughterhouse
Postures	
Standing	Supports body with limbs and abdomen not touching the floor
Lying	Places abdomen in the floor and stretch both hind legs

### 2.7 Data Collection

The behavior was recorded as soon as the JBC were tied and settled in their designated stalls. Behavioral observations lasted for 8 h, while postural observations continued for 18 h, as this reflects the average lairage time of the JBC in the slaughter facility. The videos were recorded using four cameras fixed on the walls of the slaughter facility.

### 2.8 Sampling Method

Focal sampling was used as a sampling rule in this study. This method was used in observing animal behavior where the observer selects an individual as the focal point of observation. However, for the recording rule, one–zero sampling was used to observe the eating and drinking behavior of the JBC. This technique used to record the occurrence or absence of a specific behavior. Whereas instantaneous sampling was utilized for resting, ruminating, other behaviors, ground sniffing, allogrooming and self‐grooming. This recording rule is a behavioral observation method used to record the behavior of the animals at predetermined time intervals. Meanwhile, continuous recording was used to analyze the postures (standing and lying) of the animal. This method ensures a complete and accurate account of the postural behavior patterns facilitating detailed analysis and interpretation.

### 2.9 Meat Quality Grading

Important meat quality parameters such as quality grade, dressing percentage as well as beef marbling and color were collected and analyzed for this study. The meat quality grading used a standardized method provided by the Japan Meat Grading Association. According to the guidelines, grading was determined at the 6th and 7th rib cross‐sections. Quality grade was scored from 1 to 5, with 5 indicating the highest quality. Meanwhile, marbling was evaluated using the Beef Marbling Standard (BMS) on a scale of 1 to 12, where higher values indicate more abundant marbling. For color, carcasses were graded using the Beef Color Standard, ranging from 1 to 7, with higher values indicating a darker meat color. Dressing percentage was computed by dividing the carcass weight by the live weight, multiplied by 100.

### 2.10 Statistical Analysis

All statistical analyses were conducted using RStudio (version 12.1). For count data, including all observed behaviors, generalized linear models (GLMs) were fitted using a negative binomial distribution with a log‐link function to account for overdispersion. In these models, the behavioral parameters served as the response variables, while the methods of transportation and production farms were treated as categorical fixed effects. The production farms were nested within the methods of transportation to account for the hierarchical structure of the data. Residuals were assessed graphically using the DHARMa package to ensure the validity of model assumptions.

For meat quality parameters, such as quality grade, beef marbling score, and beef color score, cumulative link models (CLMs) with a log‐link function were employed to analyze the ordinal response variables. The dressing percentage, a continuous variable, was modeled using a GLM with a Gaussian distribution and an identity link function, as this is appropriate for continuous data. In these analyses, the methods of transportation and production farms were also treated as categorical fixed effects. Likelihood ratio tests were used to compare nested models and determine the overall significance of predictors. Results were considered significant at a *p*‐value of < 0.05.

## 3 Results

### 3.1 Behavioral Parameters

Mean frequency of behaviors of each production farms between different transportation methods were shown in Table 3. There is a significant difference observed in the drinking behavior (*p* = 0.005) of JBC under different transportation methods. Higher average frequencies were observed in cattle from MF compared with those transported from the SF group. Concurrently, there is also a significant effect of transportation method in the eating behavior (*p* = 0.005) of JBC, same trends were observed where JBC from MF display higher frequency.

**TABLE 3:** Mean frequency behaviors of JBC under different transportation methods.

	Drinking	Eating	Ruminating	Resting	Others	Ground Sniffing	Allogrooming	Self‐grooming
Farm A								
Same farm (*n* = 7)	42 ± 6.77	61 ± 13.7	22 ± 7.61	112 ± 24.28	303 ± 28.41	45 ± 11.56	4 ± 1.84	2 ± 1.11
Multiple farm (*n* = 7)	97 ± 25.3	83 ± 23.8	21 ± 11.99	160 ± 33.95	209 ± 44.97	37 ± 12.94	3 ± 1.23	9 ± 5.56
Farm B								
Same farm (*n* = 7)	56 ± 12.1	31 ± 13.8	62 ± 33.49	206 ± 38.03	198 ± 47.47	26 ± 15.53	2 ± 1.1	46 ± 32.42
Multiple farm (*n* = 7)	120 ± 20.7	93 ± 10.6	26 ± 8.97	169 ± 35.49	261 ± 36.1	6 ± 3.56	1 ± 0.3	1 ± 0.46
Farm C								
Same farm (*n* = 7)	57 ± 13.0	53 ± 19.5	49. ± 32.96	217 ± 39.64	189.43 ± 39.15	117 ± 66.06	1 ± 0.59	10 ± 4.6
Multiple farm (*n* = 7)	106 ± 11.1	70 ± 8.28	13 ± 5.21	196 ± 44.17	217 ± 23.14	14 ± 7.44	2 ± 0.1	2 ± 1.03
CI	[−1.28, −0.22]	[−1.86, −0.33]	[−0.67, 2.44]	[−0.41, 0.81]	[−0.84–0.29]	[−0.46, −3.48]	[−0.5, 3.56]	[1.86, 6.12]
*p*‐value	0.005**	0.005**	0.24	0.53	0.34	0.11	0.15	0.002**

*Note:* Values are presented as means ± standard deviation. Sample size for each farm denoted by *n*. *p*‐values followed by asterisk (*) are significantly different (*p* < 0.05). ***p < 0.001; **p < 0.01; *p < 0.05.

Abbreviation: CI, confidence interval.

Although significant differences were not observed, pronounced rumination behavior was noted during the first 8 h in JBC transported from the SF method the slaughter facility.

Distinctly higher values were also observed for self‐grooming (*p* = 0.002) in cattle from Farm B transported from the SF method. Meanhwile, no clear differences were found in resting, other behaviors, ground sniffing, and allogrooming. In this study, the standing and lying positions of JBC in the slaughterhouse were observed for 18 h after transportation prior to the slaughtering procedure. There were no significant differences were found in either postural positions (Table 4) between different transportation methods. Moreover, lying positions were not observed in three cattle from the SF, nor in three cattle transported from MF.

**TABLE 4:** Mean frequency of postural behavior of BJC under different transportation methods.

	Standing	Lying
Farm A		
Same farm (*n* = 7)	896 ± 31.12	203 ± 19.24
Multiple farm (*n* = 7)	955 ± 33.51	126 ± 34.57
Farm B		
Same farm (*n* = 7)	918 ± 63.3	162 ± 63.3
Multiple farm (*n* = 7)	958 ± 52.97	122 ± 52.97
Farm C		
Same farm (*n* = 7)	893 ± 71.9	188 ± 71.9
Multiple farm (*n* = 7)	925 ± 37.35	155 ± 37.35
CI	[0.18–0.1]	[−0.65, 1.15]
*p*‐value	0.56	0.58

*Note:* Values are presented as means ± standard deviation. Sample size for each farm denoted by *n*. *p*‐values followed by asterisk (*) are significantly different (*p* < 0.05). ***p < 0.001; **p < 0.01; *p < 0.05.

Abbreviation: CI, confidence interval.

### 3.2 Meat Quality Parameters

There were no significant differences with all meat quality parameters (Table 5) measured in this study such as quality grade, dressing percentage as well as beef marbling and color.

**TABLE 5:** Meat quality parameters of the JBC under different transportation methods.

Meat quality parameters	Same farm			Multiple farm			*p*
	Farm A (*n* = 7)	Farm B (*n* = 7)	Farm C (*n* = 7)	Farm A (*n* = 7)	Farm B (*n* = 7)	Farm C (*n* = 7)	
Meat grade (%)							1
A3	—	14.3	—	—	14.3	14.3	
A4	28.6	42.8	14.3	28.6	42.8	14.3	
A5	71.4	42.9	85.7	71.4	42.9	71.4	
Dressing percentage (%)	62.3 ± 0.32	63.4 ± 0.79	62.8 ± 0.49	62.2 ± 0.56	61.8 ± 1.33	63.1 ± 0.47	0.14
Beef marbling	9 ± 0.76	7.43 ± 1.04	8.86 ± 0.59	7.86 ± 0.46	7.14 ± 0.8	9 ± 1.07	0.89
Beef color	3.71 ± 0.18	3.71 ± 0.18	3.57 ± 0.2	4.14 ± 0.26	4 ± 0.31	3.57 ± 0.2	0.39

*Note:* Values are presented as means ± standard deviation. Sample size for each farm denoted by *n*. *p*‐values followed by asterisk (*) are significantly different (*p* < 0.05). ***p < 0.001; **p < 0.01; *p < 0.05.

## 4 Discussion

This study revealed that MF groups who endured longer distances and durations as well as encountering unfamiliar animals in the transport vehicle exhibited significantly higher drinking and eating behaviors compared with animals in SF group. These observations were similar with the observation of Jarvis et al. (1996) wherein cattle transported from livestock markets who experienced extended periods of transport, additional handling and encountering unknown animals spent more time drinking during the initial hours post‐transport compared to those transported directly from farms to slaughter facility. It has also been shown that under long‐distance conditions, cattle exhibit increased feeding behavior, spending more time at the feed bunk and showing greater motivation to eat post‐transport (Ross et al. 2016; Schwartzkopf‐Genswein et al. 2007). Moreover, increased drinking and eating behavior were possibly linked to the addition of unfamiliar steer inside the vehicle, which could cause psychological stress which is an animal welfare concern. Supporting this, Wilcox et al. (2013) found that calves with unfamiliar animals significantly increased their glucose consumption compared to those that did not experience such conditions.

Self‐grooming was observed at low frequencies regardless of the transportation method; however, a significant difference was found in this behavior. JBC from Farm B in a SF group, displayed the highest frequency of self‐grooming compared with all other groups. These behaviors were regarded as essential in cattle, as Bolinger et al. (1997) found that after being restraint, animals exhibited an increase in grooming behaviors. They also observed that grooming was one of the first behaviors to occur after release, highlighting its importance as a behavioral need. Moreover, Ewing et al. (1999) stated that the suppression of normal grooming behaviors may result in the emergence of abnormal behaviors. Meanwhile, other groups in this study manifest decrease in these behaviors, as it often decreases in animals with allostatic loads and experiencing imbalance because their bodies redirect the energy from nonessential activities, like grooming, to support the immune system (Valadez‐Noriega et al. 2022). Therefore, grooming behaviors such as self‐grooming are regarded as positive welfare marker, and inhibition of this natural behavior may signal compromised welfare. However, this finding limits the generalizability of the results, as the increased in self‐grooming was observed only in cattle from Farm B in the SF group. This presents an interesting avenue for further research that considers farm‐level factors, such as management practices, to better understand the underlying causes of this behavior.

There were no significant differences were found in rumination between SF and MF groups. Older animals, like the steers in this study, may have already developed greater resilience to transport‐related stress. As the information from National Livestock Breeding Center in Japan showed that all of the JBC in this study had already experienced transportation prior to the delivery to the slaughter facility. This prior experience may have exposed the animals to transport‐related conditions, allowing them to adjust and maintain their rumination activity. On the other hand, Schwartzkopf‐Genswein et al. (2007) reported that rumination behavior was disrupted in all calves transported over both short and long distances to the feedlot. Kent and Ewbank (1990) observed a decrease in rumination time in three‐month‐old calves after 18 h of transportation. Furthermore, Schirmann et al. (2011) observed a reduction in this behavior following the addition or removal of individuals from a group (Hubbard et al. 2021; Birhanu 2020). These findings indicate that younger animals are more affected by changes in rumination after transport, likely because they feel more stress and fatigue from the journey (Marcato et al. 2020; Roadknight et al. 2021; Goetz and Renaud 2024).

Similarly, no significant differences were found in postural positions between SF and MF, but a number of previous observations have demonstrated differences in postures. Meléndez et al. (2020) revealed that 36‐h transportation resulted in a higher percentage of lying behavior in beef calves. Similarly, the steers transported for 18 h preferred to lie down after returning to their home pens (Heiderscheit et al. 2022). This tendency to lie down is likely a result of greater muscle fatigue due to the longer transport duration compared to the present study. Meanwhile, Grigor et al. (2001) found minimal lying behavior in calves transported for 9 h, even when sufficient space was provided. Some animals may have inhibited lying, as seen in this study, possibly due to the slaughter facility presenting a novel environment.

There were no significant differences were found in any of the parameters of meat quality. These findings are consistent with previous investigations that showed no significant impact on meat quality after transportation (María et al. 2003; Jones et al. 1988). However, numerous studies proved that pre‐slaughter or transportation stress have a negative impact on the meat quality that resulted to had dark, firm and dry (DFD) meat (Frimpong et al. 2014). In addition, some studies have revealed that animals transported over longer distances tend to produce less tender beef and compromise its sensory quality even though under optimal conditions (Villarroel et al. 2003; Hultgren et al. 2022). The negative impacts reported in these studies are likely attributable to longer transport durations and other contributing factors.

Several important behaviors, widely recognized as indicators of animal welfare were significantly altered such as drinking, eating and self‐grooming. This underscores the substantial impact of transportation methods on the behavioral responses of cattle. These findings contribute to a deeper understanding of animal well‐being, particularly among those transported in the MF groups. Moreover, they are vital for ensuring compliance with welfare standards and for enhancing management strategies in modern beef production systems, especially during transport. Given the complexity and importance of the transport phase, further research is warranted. Future investigations should integrate farm‐level factors, physiological stress indicators, and economic analyses to develop a more holistic understanding of transportation impacts.

## Conflicts of Interest

The authors declare no conflicts of interest.
